# Mixed integer programming with dose‐volume constraints in intensity‐modulated proton therapy

**DOI:** 10.1002/acm2.12130

**Published:** 2017-07-06

**Authors:** Pengfei Zhang, Neng Fan, Jie Shan, Steven E. Schild, Martin Bues, Wei Liu

**Affiliations:** ^1^ Department of Radiation Oncology Mayo Clinic Scottsdale AZ USA; ^2^ Department of Systems & Industrial Engineering University of Arizona Tucson AZ USA; ^3^ Department of Biomedical Informatics Arizona State University Phoenix AZ USA

**Keywords:** dose‐volume constraints, intensity‐modulated proton therapy, mixed‐integer programming

## Abstract

**Background:**

In treatment planning for intensity‐modulated proton therapy (IMPT), we aim to deliver the prescribed dose to the target yet minimize the dose to adjacent healthy tissue. Mixed‐integer programming (MIP) has been applied in radiation therapy to generate treatment plans. However, MIP has not been used effectively for IMPT treatment planning with dose‐volume constraints. In this study, we incorporated dose‐volume constraints in an MIP model to generate treatment plans for IMPT.

**Methods:**

We created a new MIP model for IMPT with dose volume constraints. Two groups of IMPT treatment plans were generated for each of three patients by using MIP models for a total of six plans: one plan was derived with the Limited‐memory Broyden–Fletcher–Goldfarb–Shanno (L‐BFGS) method while the other plan was derived with our MIP model with dose‐volume constraints. We then compared these two plans by dose‐volume histogram (DVH) indices to evaluate the performance of the new MIP model with dose‐volume constraints. In addition, we developed a model to more efficiently find the best balance between tumor coverage and normal tissue protection.

**Results:**

The MIP model with dose‐volume constraints generates IMPT treatment plans with comparable target dose coverage, target dose homogeneity, and the maximum dose to organs at risk (OARs) compared to treatment plans from the conventional quadratic programming method without any tedious trial‐and‐error process. Some notable reduction in the mean doses of OARs is observed.

**Conclusions:**

The treatment plans from our MIP model with dose‐volume constraints can meet all dose‐volume constraints for OARs and targets without any tedious trial‐and‐error process. This model has the potential to automatically generate IMPT plans with consistent plan quality among different treatment planners and across institutions and better protection for important parallel OARs in an effective way.

## 1 INTRODUCTION

Proton therapy, which has been used to treat cancer since the 1950s, offers many clinical advantages compared with the conventional radiation therapy, such as x rays or electron beams. Compared with intensity‐modulated radiation therapy (IMRT), intensity‐modulated proton therapy (IMPT) can deliver highly conformal dose distributions to the target yet spare organs at risk (OARs). Therefore, IMPT can greatly reduce the risk of OAR damage and increase the chance of local control of cancer.^1^, ^2^

In treatment planning for IMPT, we aim to deliver the prescribed dose to the target yet minimize the dose to adjacent OARs. Dose‐volume constraints are used in most modern, commercial treatment planning systems, such as Raystation (RaySearch Laboratories), Eclipse (Varian Medical Systems), and Pinnacle (Philips Radiation Oncology Systems, Philips Healthcare), to generate clinically acceptable treatment plans. These treatment planning systems all use quadratic programming models for optimization and many reports have described their use in radiotherapy. Wu and Mohan^3^ implemented soft dose‐volume constraints by quadratic programming, with the dose‐volume constraints in the objective function. Falkinger et al.^4^ proposed a prioritized optimization algorithm for IMPT planning. Gradient‐based methods^3^ and a genetic algorithm^5^ have also been used to generate treatment plans. Xing et al.^6^ reported a method for estimating the parameters for the nonlinear objective function and the corresponding algorithm used to determine those parameters. Chen et al.^7^ developed a database‐generation procedure for IMPT treatment planning.

These methods all use soft constraints and require input of initial optimization parameters, for example, penalty weights, to achieve the best balance between tumor coverage and normal tissue protection. As a result of the soft constraints applied in those methods, the desired dose volume constraints may not be rigidly satisfied in the generated results. In addition, the parameters they used are determined based on the planner's experience. Therefore, many time‐consuming trial‐and‐error iterations are needed to get the optimal initial optimization parameters. Ferris et al.^8^ showed that trial‐and‐error methods for IMRT are impractical because of the complexity of treatment planning and that use of mixed integer programming (MIP) is a more efficient method to ensure that the treatment plan meets the clinical requirements. In addition, hard constraints used in linear programming minimize the variation in plan quality among different treatment planners and across institutions, which is important for multiple institution clinical trials.

Linear programming can model clinic requirement in a mathematical model by linear relationships. Linear programming is a special case of mathematical programming, which is to optimize a linear objective function, subject to linear equality and linear inequality constraints. Additionally, we need to introduce integer variables to deal with dose volume constraints since patients’ geometry is voxelized. Therefore, mixed integer programming is a very good way for IMPT problem. Compared to quadratic programming, we can use linear programming to explicitly implement hard constraints for IMPT with the cost of complexity of models and more extensive computation time. Many investigators have studied the use of MIP in radiotherapy. For example, Cao et al.^9^ used MIP for optimizing the beam angle to deal with the uncertainties in various scenarios. To improve the plan quality in IMPT, Zaghian et al.^10^ proposed an iterative approach that could satisfy the dose‐volume constraints in MIP. Tuncel et al.^11^ derived strong inequalities for MIP in IMRT. Rocha et al.^12^ proposed a programming approach for IMRT using binary integers and obtained a plan with improved quality. Romeijn et al.^13^ proposed an MIP model for IMRT, in which the dose‐volume constraints are explicitly enforced. However, scant research exists regarding how to apply MIP to IMPT treatment planning with dose‐volume constraints (except Zaghian et al^10^).

In this study, we propose a new method to apply the MIP model with dose‐volume constraints to the IMPT treatment planning. In the MIP model, we can specify hard dose volume constraints on tumors and OARs. The generated treatment plan can satisfy all the dose volume constraints for tumors and OARs if any feasible solution exists.

In our MIP model we will specify the dose volume constraints for tumors and OARs in the optimization. If the tumor constraints (tumor coverage and homogeneity) are too stringent, there may not be a feasible solution. On the other hand, if the tumor constraints are too loose, the resultant plan is not optimal in tumor dose distribution even though it meets all the requirements for all normal tissue protection. Therefore, given the same normal tissue constraints, it is important to find the best tumor dose volume constraint parameters in MIP. Again one could use trial‐and‐error process to get as good parameters as possible. However, it is time consuming and the best result is not guaranteed. In this work, we have further developed a new method to automatically find the best parameters of tumor dose volume constraints in MIP to solve this problem.

## 2 MODEL AND FORMULATION

### 2.A Patient data and beam configurations

We retrospectively generated IMPT plans for three patients: one pediatric patient with prostate rhabdomyosarcoma (prescription dose, 45 Gy) and two patients with head and neck (H&N) cancer (prescription dose, 60 Gy or 68 Gy). These three cases are typical clinic cases, and in practice it is hard to generate good plans to meet all the dose volume constraints for these three cases due to the close proximity of critical organs to tumors. We have chosen them to demonstrate the effectiveness of our model. The corresponding dose‐volume constraints used in our clinic are shown in Table 1. The dose covering a percentage of the structure's volume (D_%_) derived from the structure's dose‐volume histogram (DVH) was compared. The planning target volume (PTV) was formed by uniform expansion of the clinical target volume (CTV) by 5 mm for the prostate rhabdomyosarcoma case and by 3 mm for the H&N cases. The PTV D_95%_ and D_5%_–D_95%_ were used to assess tumor dose coverage and homogeneity, respectively. Three beams were used for all three patients. The number of beamlets in each structure is also included in Table 1. A resolution of 5 mm was used in the dose calculation and optimization.

**Table 1:** Dose‐volume constraints

Structure	Dose volume constraints (Gy[RBE])	Achieved values from MIP (Gy[RBE])	Achieved values from L‐BFGS (Gy[RBE])
Prostate (with 3837 beamlets)			
Planning target volume D_95%_	>44	44	44
Planning target volume D_5%_–D_95%_	<2	1.6	2
Bladder D_25%_	<45	44.2	44.5
Femoral heads D_10%_	<28	12.4	14.1
Rectum D_25%_	<45	45	44.8
H&N, case 1 (with 7773 beamlets)			
Planning target volume D_98%_	>59	60	59.3
Planning target volume D_2%_–D_98%_	<2	0	1.3
Whole brain D_1%_	<62	60	59.5
Brain stem D_1%_	<55	55	53
Optic chiasm D_1%_	<55	55	56.1
H&N, case 2 (with 9905 beamlets)			
Planning target volume D_98%_	>67	68	67.6
Planning target volume D_2%_–D_98%_	<2	0	0.8
Brain stem D_1%_	<55	25.2	24.3
Mucosa avoid D_1%_	<30	15.4	16.3
Spinal cord D_1%_	<45	20.2	21.9

For each patient, we used two optimization methods to generate IMPT plans with identical dosimetric goals: the MIP model and quadratic programming method. The optimization software, IBM CPLEX (version 12.5)^13^ was used to solve the MIP problems. We also generated IMPT plans for the same patients using a quadratic programming model solved by the Limited‐memory Broyden–Fletcher–Goldfarb–Shanno (L‐BFGS) method. L‐BFGS is a limited‐memory quasi‐Newton code for unconstrained optimization. L‐BFGS is similar to the classical gradient descent method, but it uses some approximation to minimize the memory overhead. Therefore it is particularly well suited for optimization problems with a large number of variables. IMPT treatment planning involves a huge number of variables (usually as large as 50,000), thus it is a good application for L‐BFGS. Currently the new version of the commercial treatment planning system, Eclipse^TM^, has an option to use L‐BFGS as an optimizer. Thus we believe that it is a good idea to benchmark our new method with the L‐BFGS method. Please note that we used the same beamlets (influence matrix) for both quadratic programming and MIP for fair comparison.

### 2.B Implementation of dose‐volume constraints

For each voxel i within each structure $I\in \left\{\mathrm{OAR},T\right\}$ (tumors are denoted as T), $D_{0}^{I}$ and $D_{i}^{I}$ denote the prescribed dose value and the plan dose, respectively, and P^I^ is the percentage of the structure volume covered by a certain prescribed dose $D_{0}^{I}$. In addition, N^I^ denotes the total number of voxels of the structure I, and $D_{0}^{\mathrm{TL}}$ (upper) and $D_{0}^{\mathrm{TR}}$ (lower) are the prescribed doses covering the corresponding $P_{∼}^{\mathrm{TL}}$ (upper) and $P_{∼}^{\mathrm{TR}}$ (lower) percentage of the volume of the target, respectively. For example, by specifying the $P_{∼}^{\mathrm{TR}}$ to be 0.95, the $D_{0}^{\mathrm{TR}}$ becomes D_95%_. The binary decision variables $y_{i}\in \left\{0,1\right\}$ denote whether the dose at the voxel i is less than the prescribed dose value (y_i_ = 0) or not (y_i_ = 1), and $y=\left(y_{1},y_{2},\;\ldots \;,y_{n}\right)$ denotes the binary decision variables for all voxels (i = 1, 2, … , n).

The dose‐volume constraints are specified for normal organs or for hot spots and cold spots in tumors. The dose‐volume constraints limit the percentage of the structure volumes with doses exceeding the prescribed dose value to be less than or equal to the specified value of $P_{∼}^{I}$ or $P_{∼}^{\mathrm{TR}}$. In addition, to ensure the specified dose‐volume constraints are met for cold spots in tumors, we limit the percentage of the tumor volumes that receive lower doses than the prescribed dose value to be less than or equal to the specified value of $P_{∼}^{\mathrm{TL}}$. The MIP formulation follows (called ***Model 1*** hereafter),

(1a) $$\min\sum_{i\in T}\left|D_{i}^{T}-D_{0}^{T}\right|$$

(1b) $$\text{s.t.}\;D_{i}^{I}\leq D_{0}^{I}+My_{i}^{I},\forall i\in I,\forall I\in OAR,$$

(1c) $$D_{i}^{I}\geq D_{0}^{I}y_{i}^{I},\forall i\in I,\forall I\in OAR,$$

(1d) $$\sum_{i\in T}y_{i}^{I}/N^{I}\leq P^{I},\forall I\in OAR,$$

(1e) $$D_{i}^{T}\leq D_{0}^{TR}+My_{i}^{TR},\forall i\in T,$$

(1f) $$D_{i}^{T}\geq D_{0}^{TR}y_{i}^{TR},\forall i\in T,$$

(1g) $$\sum_{i\in T}y_{i}^{TR}/N^{T}\leq P^{TR},$$

(1h) $$D_{i}^{T}\leq D_{0}^{TL}+M\left(1-y_{i}^{TL}\right),\forall i\in T,$$

(1i) $$D_{i}^{T}\geq D_{0}^{TL}\left(1-y_{i}^{TL}\right),\forall i\in T,$$

(1j) $$\sum_{i\in T}y_{i}^{TL}/N^{T}\leq P^{TL},$$

(1k) $$D_{i}^{T}=\sum_{j}w_{i,j}^{T}x_{j}^{},\forall i\in T$$

(1l) $$D_{i}^{I}=\sum_{j}w_{i,j}^{I}x_{j}^{},\forall i\in I,\forall I\in OAR$$

(1m) $$y_{i}^{I}\in \left\{0,1\right\},\forall i\in I,\forall I\in OAR,$$

(1n) $$y_{i}^{TL},y_{i}^{TR}\in \left\{0,1\right\},\forall i\in T$$

(1o) $$x_{j}^{}\geq 0,\forall j\in J$$

where M is a sufficiently large positive number. The role of M is to make the inequality hold when the term associated with M takes a value of 1 (i.e., *y*_*i*_ = 1). We can choose M to be the upper bound of all possible $D_{0}^{I}$ for OARs and $D_{0}^{TR}$ for targets. For the cases included in this research, it is set to be 100 Gy[RBE]. It is worth noting that it is better not to set M too large, because as M becomes larger, all the constraints, which contain M, become less contingent, and thus the computation time becomes longer. The objective (1a) is to minimize the total dose deviation on tumors. Instead of the quadratic objective function, we used the absolute value to compute total dose deviation. Constraints (1b) and (1c) together are to decide whether the dose at a voxel *i* of a normal organ *I* exceeds the prescribed dose *D*_0_ or not. If the dose at a voxel *i* of a normal organ *I* does not exceed the prescribed dose *D*_0_, then the corresponding binary variable *y*_*i*_ is 0. Otherwise, the corresponding binary variable *y*_*i*_ is 1. This is facilitated by a sufficiently large positive number *M*. The role of M is to ensure that the constraints hold when y variable is 1. Constraints (1d) are the dose‐volume constraints for OARs with the help of the binary variable *y*_*i*_, determined by (1b) and (1c). We limit the percentage of the organ *I* volumes with doses exceeding $D_{0}^{I}$ to be less than or equal to *P* ^*I*^. Similarly, constraints (1e) to (1g) are the dose‐volume constraints for tumor hot spots. We limit the percentage of the tumor volumes with doses exceeding $D_{0}^{TR}$ to be less than or equal to *P* ^*TR*^. Constraints (1h) to (1j) are for the dose‐volume constraints for tumor cold spots. Equations. (1k) and (1l) represent the total dose to each voxel from all beamlets, and *x*_*j*_ are continuous variables representing the *j*th beamlet intensity (fluence), while the influence matrix, *w*_*i*,*j*_, is the dose contribution of the beamlet *j* of unit intensity at voxel *i*. The influence matrix is precalculated using an in‐house developed IMPT dose calculation engine.^14^, ^15^ Constraints (1m) and (1n) are the constraints for the binary decision variables.

Note that in the objective function, we only include terms for tumors but no terms for OARs, this is because all desired constraints for OARs are included in the constraints in the model (unlike quadratic programming, the dose volume constraints are usually included in the objective function as “soft constraints”). Note that in our model, we have products of continuous variables and binary variables. This kind of nonlinear terms can be linearized by introducing one additional continuous variable and additional constraints. For example, we can linearize $D_{0}^{I}y_{i}^{I}$ by introducing variable $z_{i}^{I}$, constant M (large enough) and following constraints:

$$z_{i}^{I}\geq D_{0}^{I}y_{i}^{I},z_{i}^{I}\leq D_{0}^{I},z_{i}^{I}\geq 0,z_{i}^{I}\leq My_{i}^{I}$$

We have modeled all requirements as hard constraints. In practice, this can ensure all the dose volume constraints. Usually, target coverage may be satisfied to satisfy constraints for some critical organs such as spinal cord and brain stem. In such situation, we can always change the parameters in the constraints to achieve the best balance with the “trial‐and‐error” procedure.

### 2.C Minimizing the trial‐and‐error procedure

There is a drawback to our MIP model with dose volume constraints. After we set the dose‐volume constraints for structures, the original optimization problem (Model 1) might not have feasible solutions. We have to try different values for D_5%_ and D_95%_ to find the best balance between target coverage and protection of OARs, which can ensure good homogeneity. If the values are too stringent, Model 1 becomes infeasible; if the values are too loose, the resulting plan is not optimal for the tumor dose distribution. Therefore, it is important to find ways to obtain the best values of $D_{0}^{\mathrm{TR}}$ and $D_{0}^{\mathrm{TL}∼}$so that we obtain the optimal balance.

One potential way to acquire optimal parameters is to use the trial‐and‐error procedure by solving Model 1 with different values for $D_{0}^{\mathrm{TR}}$ and $D_{0}^{\mathrm{TL}}$; however, this procedure is tedious and optimal results are not guaranteed. In this section, we propose the following method to find the best parameters for tumors given the same dose volume constraints for normal tissues. In the following explanation, we are going to take the prostate case as an example. If $P^{\mathrm{TR}}=P^{\mathrm{TL}}=0.05$, the $D_{0}^{\mathrm{TR}}$ becomes D_95%_, and the $D_{0}^{\mathrm{TL}}$ becomes D_5%_. We propose a new way to obtain the best parameters more efficiently (called ***Model 2*** hereafter) to minimize the trial‐and‐error process as follows,

(2a) $$\min d_{r}^{T}-d_{l}^{T}$$

(2b) $$\text{s.t.}\;D_{i}^{I}\leq D_{0}^{I}+My_{i}^{I},\forall i\in I,\forall I\in OAR,$$

(2c) $$D_{i}^{I}\geq D_{0}^{I}y_{i}^{I},\forall i\in I,\forall I\in OAR,$$

(2d) $$\sum_{i\in I}y_{i}^{I}/N^{I}\leq P^{I},\forall I\in OAR,$$

(2e) $$D_{i}^{T}\leq d_{r}^{T}+My_{i}^{TR},\forall i\in T,$$

(2f) $$D_{i}^{T}\geq d_{r}^{T}y_{i}^{TR},\forall i\in T,$$

(2g) $$\sum_{i\in T}y_{i}^{TR}/N^{T}\leq P^{TR},$$

(2h) $$D_{i}^{T}\leq d_{l}^{T}+M\left(1-y_{i}^{TL}\right),\forall i\in T,$$

(2i) $$D_{i}^{T}\geq d_{l}^{T}\left(1-y_{i}^{TL}\right),\forall i\in T,$$

(2j) $$\sum_{i\in T}y_{i}^{TL}/N^{T}\leq P^{TL},$$

(2k) $$D_{i}^{T}=\sum_{j}w_{i,j}^{T}x_{j}^{},\forall i\in T$$

(2l) $$D_{i}^{I}=\sum_{j}w_{i,j}^{I}x_{j}^{},\forall i\in I,\forall I\in OAR$$

(2m) $$y_{i}^{I}\in \left\{0,1\right\},\forall i\in I,\forall I\in \left\{OAR,T\right\},$$

(2n) $$y_{i}^{TL},y_{i}^{TR}\in \left\{0,1\right\},\forall i\in T$$

(2o) $$x_{j}^{}\geq 0,\forall j\in J$$

The $d_{l}^{T}$ and $d_{r}^{T}$ are the new decision variables for the dose‐volume constraints of the cold and hot spots for tumors, respectively. We minimize their difference to ensure that we achieve the best balance. The objective (2a) is to minimize the difference between the dose of the cold and hot spots for tumors. Constraints (2b) and (2c) together are to decide whether the dose at a voxel *i* of a normal organ *I* exceeds the prescribed dose *D*_0_ or not. If the dose at a voxel *i* of a normal organ *I* does not exceed the prescribed dose *D*_0_, then the corresponding binary variable *y*_*i*_ is 0. Otherwise, the corresponding binary variable *y*_*i*_ is 1. This is facilitated by a sufficiently large positive number *M*. Constraints (1d) are the dose‐volume constraints for OARs with the help of the binary variable *y*_*i*_, determined by (2b) and (2c). We limit the percentage of the organ *I* volumes with doses exceeding $D_{0}^{I}$ to be less than or equal to *P* ^*I*^. Similarly, constraints (2e) to (2g) are the dose‐volume constraints for tumor hot spots. We limit the percentage of the tumor volumes with doses exceeding $d_{r}^{T}$ to be less than or equal to *P* ^*TR*^. Constraints (2h) to (2j) are for the dose‐volume constraints for tumor cold spots. Equations. (2k) and (2l) represent the total dose to each voxel from all beamlets, and *x*_*j*_ are continuous variables representing the *j*th beamlet intensity (fluence), while the influence matrix, *w*_*i*,*j*_, is the dose contribution of the beamlet *j* of unit intensity at voxel *i*. Constraints (2m) and (2n) are the constraints for the binary decision variables. Constraints (1o) enforce the non‐negativity of x variables.

## 3 RESULTS

**Figure 1:**
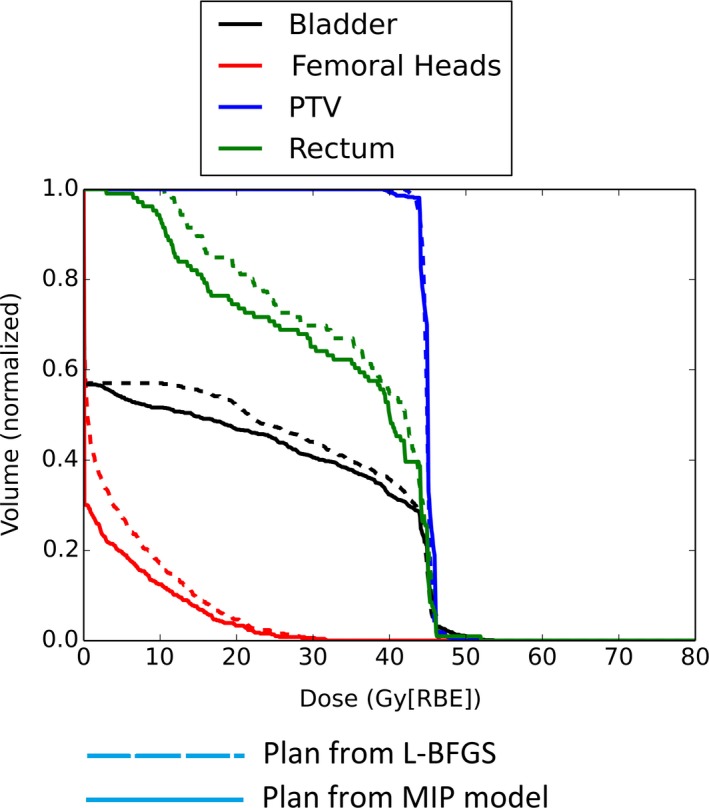
DVH for the patient 1 with pediatric prostate rhabdomyosarcoma.

**Figure 2:**
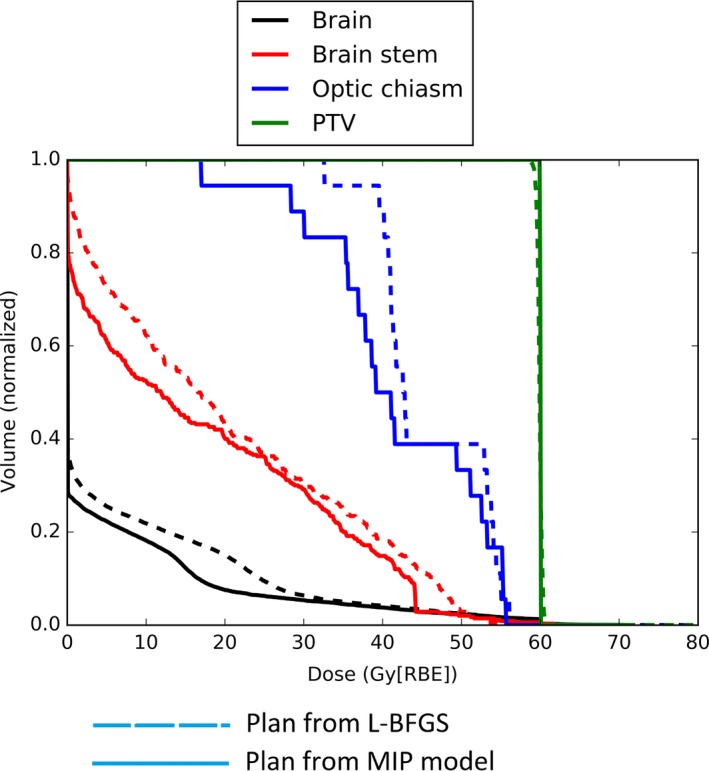
DVH for patient 2 with H&N cancer.

**Figure 3:**
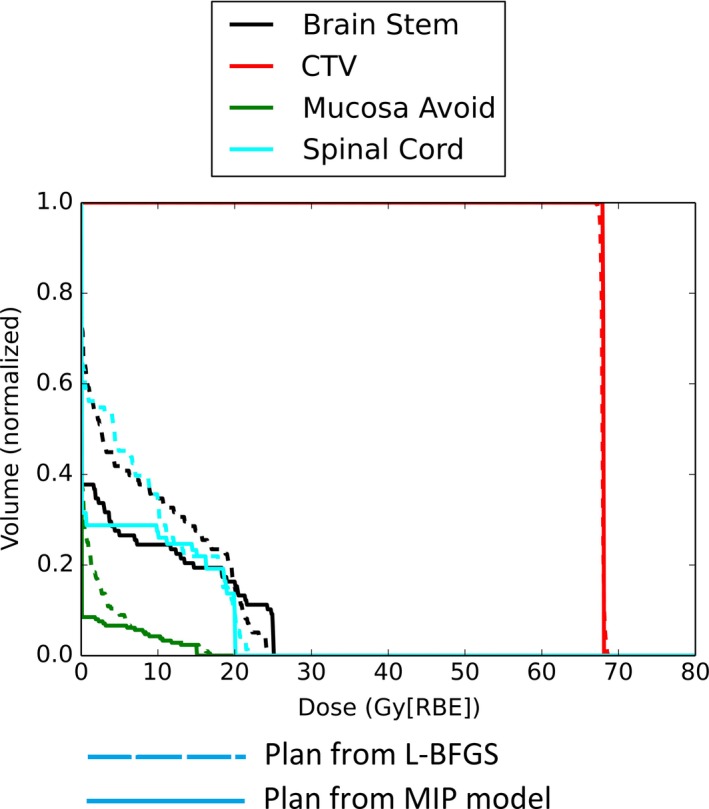
DVH for patient 3 with H&N cancer.

We have used Model 2 to determine the best parameters for all three cases. The optimal values derived are supplied to Model 1 to generate the plans for these three cases. Figure 1 shows the DVH results for the pediatric patient with prostate rhabdomyosarcoma, and Figs. 2 and 3 show the DVH results for the patients with H&N cancer. The corresponding DVH indices are also shown in Table 1 with the corresponding dose volume constraints.

From Figs. 1–3, by comparing DVH results of normal organs and tumors, we can see that our results provide comparable tumor dose coverage, tumor dose homogeneity and the maximum doses to OARs. At the same time, the mean doses of OARs are notably reduced. Another interesting observation is that the DVHs from the MIP method tend to be discontinuous, compared to the DVHs from the L‐BFGS method.

**Figure 4:**
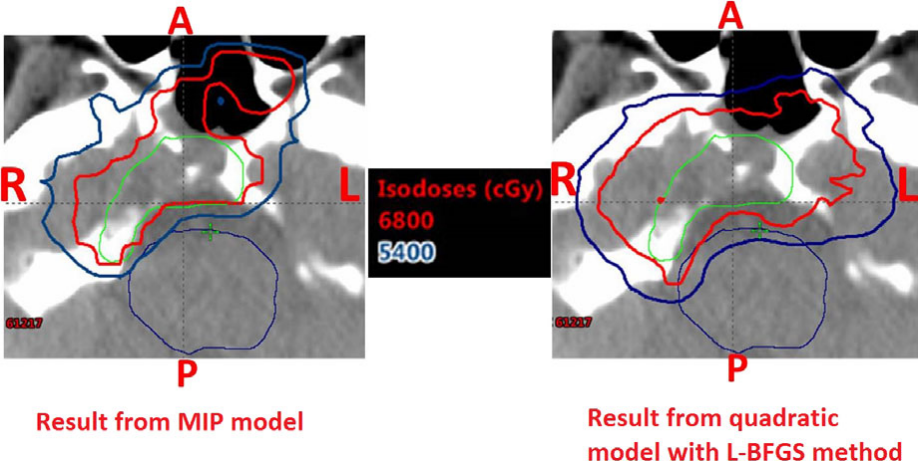
Dose distribution results at one CT slice for patient 2: MIP model with dose‐volume constraints (left) *and quadratic model solved by* L‐BFGS method (right). Green: Clinical target volume (CTV), Cyan: Brain stem. Red: prescription iso‐dose line (68 Gy[RBE]), Dark Blue: iso‐dose line of 54 Gy[RBE]. MIP model with dose‐volume constraints generated a plan with much better brain stem protection at the cost of slight tumor underdosage.

Figure 4 shows the typical result of the dose distribution at one CT slice. Green line is for the CTV, cyan line is for the brainstem, red line is for the prescription iso‐dose line (68 Gy) and dark blue line is for the iso‐dose line of 54 Gy. We can clearly see the better brainstem protection from our model. However, small part of the tumor volume is underdosed. In the prostate case, after we enforced the dose‐volume constraints in the MIP model, D_25%_ of the rectum and the bladder improved with dose‐volume constraints. However, protection of the rectum and bladder came at the cost of tumor coverage. The D_95%_ of the tumors decreased in our result from MIP model than that from the quadratic programming method. For both H&N cases, interestingly both D_98%_ and D_2%_–D_98%_ of the tumors are improved with better protections of all the normal tissues from our model.

## 4 DISCUSSION

The MIP model with dose‐volume constraints can generate treatment plans that meet all dose‐volume requirements for OARs. The new model can generate IMPT treatment plans with comparable target dose coverage, target dose homogeneity, and the maximum dose to OARs compared to treatment plans from the conventional quadratic programming method without any tedious trial‐and‐error process. Some notable reduction in the mean doses of OARs is observed.

In addition, Model 2 is very effective to find the optimal parameters for obtaining the best balance between the tumor coverage and OAR sparing. The dose‐volume constraints for the OARs are rigid especially for some important organs like spinal cord and brain stem. In some scenarios tumor coverage may have to be sacrificed slightly to meet the dose volume constraints for these important organs.

To demonstrate our method, we have set constraint parameters to certain values to show that the plans generated from our proposed method meet our institution's dose volume constraints. We can certainly modify these parameters of dose volume constraints to achieve different results to protect normal organs better or relax the requirements for normal organs’ protection to improve the tumor coverage. This is a medical decision and thus the user should be able to modify the corresponding parameters of dose volume constraints to achieve the desired plan depending on the patient specific clinical priority. Alternatively we can achieve the soft constraints in linear programming using so‐called “fuzzy logic based optimization”, which will generate the best normal tissue protection given the fixed tumor coverage without any tedious trial‐and‐error process. This is the ongoing research in our group. On the other hand, we can include soft constraints in the objective function in quadratic programming as proposed by Wu and Mohan^3^ This is the most popular way to implement the dose volume constraints in radiotherapy planning.

Generally, the maximum dose of normal tissue from the model with the dose‐volume constraints is comparable to that from the quadratic programming method. However, the mean dose of some normal tissues is remarkably reduced (see Figs. 1–3). This might be important for the sparing of some important parallel organs such as parotids, oral cavity, total lung, etc. Interestingly from Figs. 1–3 the DVHs from the MIP method tend to be discontinuous, while the DVHs from the L‐BFGS method are smooth. We speculate that this might be due to the hard constraints used in the MIP method and we believe that these insignificant discontinuities would not be clinically important.

Our MIP model only needs to run once to achieve the results, while quadratic programming method may need to run several times to get a better result (i.e., trial‐and‐error). We have done “trial‐and‐error” in quadratic programming to get the best results and compared the best results from quadratic programming with results from the MIP model. This shows the advantage of our model that the tedious “trial‐and‐error” process is not needed in our proposed method.

This advantage can be explained in more detail. The dose‐volume constraints are hard in contrast to the soft constraints used in the conventional, heuristic methods. Our method uses an accurate model that produces a better result more efficiently. Our model ensures that the dose‐volume constraints are rigidly met for both OARs and tumors. After solving our model, the optimal solution can satisfy all the dose‐volume constraints because of these hard constraints. On the other hand, the result from any heuristic algorithm cannot guarantee that all the dose‐volume constraints are met. Meeting the constraints can improve protection for OARs and thus can lead to better quality of life for patients. The proposed model can also inform the treatment planner that no optimal solution exists because of too stringent tumor dose constraints before we start to solve the problem. Heuristic methods do not have this capability. In addition, the iterations of the trial‐and‐error process used in heuristic methods are time‐consuming, and an optimal result is not guaranteed, while our second model can derive the best parameters for generating plans with the best balance between tumor coverage and protection of OARs.

We also compared the beam intensity results from our model with those from a quadratic programming model solved by the L‐BFGS method. Interestingly, the number of beamlets with nonzero intensity was much lower in our linear model than in a nonlinear model, which is consistent with other reports.^9^ Fewer beamlets with nonzero intensity will improve the delivery efficiency, which is another advantage of our linear model.

Given the dose volume constraints of normal tissues, in some situations too stringent tumor dose constraints would make the Model 1 infeasible; while in some situations too loose tumor dose constraints would lead to undesired tumor dose distribution. Therefore it is important to set proper tumor dose constraint parameters in our MIP model. We used Model 2 to determine the optimal tumor dose constraint parameters that would achieve the best balance between tumor dose coverage and OAR sparing more efficiently. In all of our example cases, Model 2 allowed a much better balance than the conventional trial‐and‐error procedure. It also turns out that Model 2 always achieves satisfactory tumor dose distributions, which meet the tumor dose‐volume constraints at least for the three clinical cases included in this study. We believe that the use of Model 2 would improve the quality of treatment plans.

Our model has certain limitations. First, a complex problem requires substantial time to reach a final, optimal solution, so we currently terminate the optimization if we have a feasible solution, whose objective value is within a certain gap tolerance compared to the optimal solution. In our examples, we can get a feasible solution with 50% gap within 2 hr in all cases (for the case of patient 3, we get the optimal result within 5 s). We believe that we can significantly improve the efficiency of our current model by using either Benders’ decomposition^16^ or parallel computing.

## 5 CONCLUSION

We have proposed a model that can generate treatment plans meeting all the requirements for dose‐volume constraints of OARs without any tedious trial‐and‐error process. This model has the potential to automatically generate IMPT plans with consistent plan quality among different treatment planners and across institutions and better protection for important parallel OARs in an effective way.

We are working toward developing more efficient algorithms that will allow us to use the model for patient population study. To incorporate robust optimization in linear programming for IMPT treatment planning is also another future research direction.

## CONFLICTS OF INTEREST

The authors declare no conflict of interest.
